# Effective use of dupilumab for eosinophilic gastritis concomitant with severe asthma

**DOI:** 10.1186/s13223-024-00940-5

**Published:** 2024-12-18

**Authors:** Tomohito Takeshige, Ryo Koyama, Hiroaki Motomura, Akifumi Okajima, Toshihiko Nishioki, Junko Watanabe, Toshifumi Yae, Kenji Kido, Kazuhisa Takahashi

**Affiliations:** 1 Department of Respiratory Medicine, Juntendo University Nerima Hospital, 3-1-1, Takanodai, Nerima-ku, Tokyo, 177-8521 Japan; 2 Department of Respiratory Medicine, Juntendo University Faculty of Medicine and Graduate School of Medicine, 3-1-3, Hongo, Bunkyo-ku, Tokyo, 113-8431 Japan

**Keywords:** Eosinophilic gastrointestinal diseases (EGIDs), Eosinophilic gastritis (EoG), Bronchial asthma, Dupilumab

## Abstract

**Background:**

Eosinophilic gastrointestinal diseases (EGIDs) are chronic immune-mediated inflammatory disorders characterized by gastrointestinal symptoms and eosinophilic inflammation in specific regions of the gastrointestinal tract. “Eosinophilic gastritis” (EoG) refers to the condition in which the stomach is involved. In patients with EoG, approved treatment options are restricted despite the high mortality associated with the condition. Dupilumab is a human monoclonal antibody directed against the interleukin (IL)-4 receptor α subunit and inhibits the signaling pathways of both IL-4 and IL-13. The real-world data on the effectiveness of dupilumab for EoG are limited. We present the case of a patient with EoG and accompanying severe asthma who demonstrated improvement with dupilumab administration.

**Case presentation:**

A 35-year-old woman who had been treated for asthma complained of worsening intermittent upper abdominal pain. Her dyspnea aggravated and she was admitted to our hospital for asthma exacerbation. Despite the improvement in her asthma symptoms with systemic corticosteroids, her abdominal pain persisted. Upper gastrointestinal endoscopic mucosal biopsy revealed eosinophilic cell infiltration; therefore, the patient was diagnosed with EoG. Dupilumab administration was initiated for asthma, while improvement of secondary EoG was expected. Following dupilumab administration, both EoG and asthma symptoms, disease control, laboratory findings, endoscopic findings, and pathological findings improved. No adverse events have been reported after the dupilumab treatment.

**Conclusion:**

This case report supports that dupilumab could be an effective treatment option for EoG and accompanying severe asthma.

## Background

Eosinophilic gastrointestinal diseases (EGIDs) are chronic immune-mediated inflammatory disorders characterized by gastrointestinal (GI) symptoms and eosinophilic inflammation in particular regions of the GI tract without known etiology of eosinophilia [1, 2]. International consensus recommendations have been established for the terminology and definitions of EGIDs [3]. The overall disease category is EGID. EGID is used as the umbrella term for GI disorders with pathologic eosinophilic infiltration without secondary causes. Eosinophilic esophagitis (EoE) is the most common among EGIDs, with an estimated prevalence of 57 cases per 100,000 people in the United States [4]. This term is indicative of esophageal involvement alone, in which eosinophil infiltration is localized only in the esophagus without involvement of any other parts of the GI tract [5]. Any other site of involvement is referred to as non-EoE EGID, which is further categorized according to the location of eosinophil inflammation, such as eosinophilic gastritis (EoG) for the stomach, eosinophilic enteritis (EoN) for the small intestine, and eosinophilic colitis (EoC) for the colon.

Non-EoE EGID is a rare primary GI disorder of unknown etiology characterized by eosinophilic infiltration observed in histopathological examination of the intestinal mucosa extending from the esophagus to the rectum [6]. Non-EoE EGID causes a wide range of GI symptoms, including abdominal pain, diarrhea, nausea, vomiting, bloating, or ascites [6]. The prevalence of non-EoE EGID is estimated to be 28 cases per 100,000 persons [7], while that of EoG is estimated at 6.3 cases per 100,000 persons [8]. Patients with non-EoE EGID experience more severe symptoms and a worse quality of life compared to those with EoE [9]. Of patients with non-EoE EGID, 46% had a history of allergy. Furthermore, bronchial asthma (27.8%) was the most commonly reported allergic disease [10]. Increased mortality was observed in patients with EoG and EoN [11].

The first-line treatment for EoE is proton pump inhibitors (PPIs). Approximately 50% of the patients exhibit histological improvements following treatment with PPI [12].

Systemic glucocorticoids are the most frequently used agents for non-EoE EGIDs. Moreover, patients may undergo spontaneous remission with oral corticosteroids. However, in 60% of patients, the disease relapses after treatment with corticosteroids, with nearly half of the patients experiencing irregular exacerbations and persistent symptoms [13]. In addition to corticosteroids, viable treatment options include leukotriene receptor antagonists, histamine H1 receptor antagonists, mast cell stabilizers, dietary therapy, and immunosuppressants. However, the efficacy of these drugs has not been confirmed [14–18]. Despite the high mortality, the approved treatment options for EoG are restricted; therefore, drug options with diverse pharmacological effects are warranted.

Dupilumab is a human monoclonal antibody directed against the interleukin (IL)-4 receptor α subunit and inhibits the signaling pathways of both IL-4 and IL-13. Dupilumab has been approved for patients with asthma [19, 20], atopic dermatitis [21], chronic sinusitis with nasal polyposis [22], prurigo nodularis [23], chronic spontaneous urticaria [24], and eosinophilic esophagitis [25]. Approval for indications varies by country. The pathophysiology of EoG entails the activation of IL-4, IL-5, and IL-13 pathways [26]. Consequently, the inhibition of IL-4 and IL-13 signaling by dupilumab may prove effective for EoG. However, real-world data evidence regarding the efficacy of dupilumab in treating EoG remains scarce.

Here, we present a case in which EoG and accompanying severe asthma improved after dupilumab administration.

## Case presentation

**Fig. 1:**
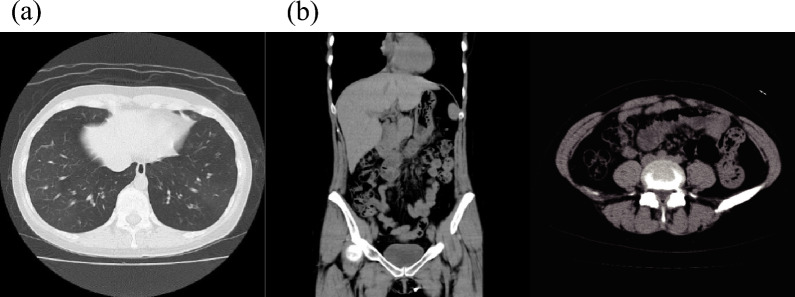
Image findings. **a** Mild bronchial thickening in chest computed tomography. **b** No ascites or intestinal wall thickening is noted

A 35-year-old woman, previously managed for asthma at a local hospital under GINA step 4 treatment for 7 years, presented to our hospital with dyspnea that commenced 1 day before admission. The diagnosis was asthma exacerbation, prompting the introduction of additional systemic corticosteroids and an inhaled short-acting beta-2 agonist, resulting in an improvement of her symptoms. The patient was discharged after 1 week of hospitalization. One month later, she visited for an unscheduled consultation due to worsening intermittent upper abdominal pain. The pain did not improve despite the use of analgesics and PPI. Around the same time, her dyspnea worsened, and she was hospitalized once again for a new asthma exacerbation. The laboratory findings from the second admission are presented in Table 1. The patient had no history of allergies to any particular food, and specific immunoglobulin (Ig) E tests for egg, milk, wheat, soybean, peanut, shrimp, and crab were negative. Chest computed tomography (CT) revealed mild bronchial thickening. However, no ascites or intestinal wall thickening was observed (Fig. 1).

**Table 1:** Laboratory findings

Hematology	
WBC	13,800/µL
Neutrophils	8073/µL
Lymphocytes	2346/µL
Eosinophils	3036/µL
Monocytes	345/µL
RBC	481 × 10^4^
Hb	14.1 g/dL
Hct	42.2%
Plt	32.2 × 10^4^
Biochemistry	
Alb	3.7 g/dL
AST	12 IU/L
ALT	8 IU/L
LDH	219 IU/L
BUN	9 mg/dL
Cre	0.67 mg/dL
Na	139 mM/L
K	4.1 mM/L
Cl	104 mM/L
Serology	
CRP	0.4 mg/dL
PR3-ANCA	<1.0 U/mL
MPO-ANCA	<1.0 U/mL
IgE	780 IU/mL
Cedar	65.8 (class4)
Timothy	7.32 (class2)

**Fig. 2:**
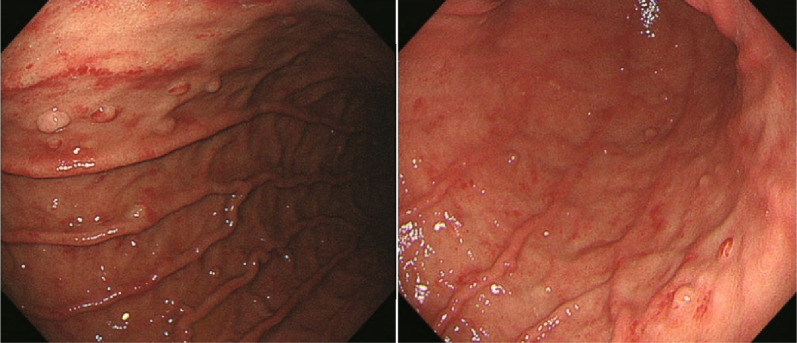
Gastrointestinal endoscopy image displaying erosive gastritis and fundic gland polyps

**Fig. 3:**
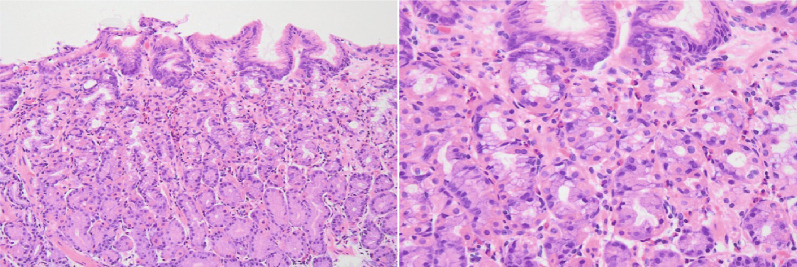
Pathological findings demonstrating increased gastric eosinophilic cell infiltration

Although asthma symptoms improved with systemic corticosteroid administration, the abdominal pain persisted. Upper GI endoscopy revealed erosive gastritis and fundic gland polyps (Fig. 2). On the other hand, examination of the esophagus and duodenum were normal. Mucosal biopsies were performed three times from the stomach. Mucosal biopsy revealed eosinophilic cell infiltration (Fig. 3). The number of eosinophils per high power field (HPF) was greater than 30 in 5 HPF, consistent with the histopathologic features of EoG [27]. Other GI disorders that cause eosinophilia were also ruled out. There were no findings suggesting Crohn's disease such as diarrhea, anal lesions, or anemia, and upper GI endoscopy did not reveal any findings characteristic of Crohn's disease. The patient did not have close contact with animals at work or as a pet. In addition, the patient did not eat raw freshwater fish, crabs, animal meat, reptiles, or organic vegetables. There were no such behaviors that increased the risk of parasitic infections. There were no findings suggestive of vasculitis, such as numbness, purpura, arthralgia, or myalgia, and PR3-ANCA and MPO-ANCA were negative, making the diagnosis of EGPA unlikely. Therefore, diagnosis of EoG was established.

The patient experienced persistent symptoms of asthma and exacerbations despite optimized maximal GINA step 5 treatment. Therefore, biological therapies were incorporated into the treatment. The score of the asthma control test (ACT) was 10, indicating poor asthma control. Thus, dupilumab administration was initiated for asthma, while a secondary improvement in the EoG was expected. Before the drug administration, the serum eosinophil count was 726/µL.

**Fig. 4:**
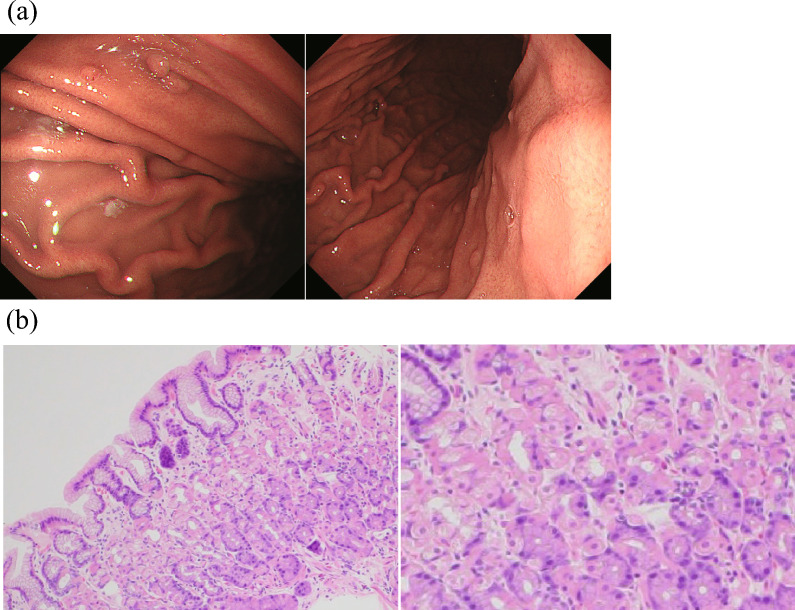
Gastrointestinal endoscopy and pathological examination findings after dupilumab administration. **a** Normal gastric mucosa without signs of gastritis. **b** Few eosinophilic cells

Sixteen weeks after dupilumab administration, the patient’s EGID symptom score decreased from 35 to 3. For asthma, ACT scores improved from 10 to 25. After 92 weeks of administration, fractional exhaled nitric oxide (FeNO) decreased from 133 parts per billion (ppb) to 14 ppb. Blood tests revealed a decrease in eosinophil count from 726/µL to 441/µL, while IgE levels decreased from 289 to 26.1 IU/mL. Endoscopic and pathological changes were observed at 184 weeks. The gastric mucosa was normal, with no signs of gastritis. Mucosal biopsies were performed two times from the stomach. In pathological examination, the number of eosinophils per HPF was 0–2 in 5 HPF (Fig. 4). No adverse events were observed after the dupilumab treatment.

## Discussion

To the best of our knowledge, this is the first case of an adult with EoG concomitant with severe asthma, in whom both EoG and asthma improved with the administration of dupilumab. To date, cases of EoE that have demonstrated improvement with dupilumab treatment have been included in clinical trials [28] while evidence of this treatment’s efficacy has been observed in real-world data [29]. However, evidence of the efficacy and safety of dupilumab in patients with EoG is limited to case reports and retrospective case series [30–33].

The biological agents for asthma, currently approved in Japan include mepolizumab, benralizumab, tezepelumab, omalizumab, and dupilumab.

Mepolizumab is a humanized monoclonal antibody that binds to and neutralizes IL-5. Additionally, mepolizumab does not improve clinical symptoms in patients with EoE [34]. Case reports have demonstrated the efficacy of mepolizumab in non-EoE EGIDs [35].

Benralizumab is an IL-5 receptor α (IL-5Rα)-directed cytolytic monoclonal antibody that does not improve clinical symptoms in patients with EoE (NCT04543409). However, a phase III trial in patients with non-EoE EGIDs is currently ongoing (NCT05251909).

Tezepelumab is a human monoclonal antibody that specifically binds to thymic stromal lymphopoietin. A phase III trial is currently underway in patients with EoE (NCT05583227). No clinical trials or case reports of non-EoE EGIDs are available.

Omalizumab is a humanized monoclonal antibody against human IgE that binds to the human FcγRIIb receptors. Clinical symptoms did not improve in patients with EoE who received omalizumab [36], while no additional progress was observed in non-EoE EGIDs in the phase III study completed in 2007 (NCT00084097).

Dupilumab is a human monoclonal antibody directed against the IL-4 receptor α subunit and inhibits the signaling pathways of both IL-4 and IL-13. Dupilumab has been approved for patients with asthma [19, 20], atopic dermatitis [21], chronic sinusitis with nasal polyposis [22], prurigo nodularis [23], chronic spontaneous urticaria [24], and eosinophilic esophagitis [25]. Additionally, dupilumab is effective for asthma exacerbations in patients with a baseline eosinophil count >150/µL or high FeNO > 25 ppb [20]. In our patient’s case, these criteria were met, allowing the initiation of dupilumab for asthma treatment. Furthermore, this drug is currently being assessed for EGID and has been approved for EoE [25]. In EoG, a phase II, double-blinded, placebo-controlled trial is currently ongoing to assess the efficacy of dupilumab (NCT03678545).

Dupilumab is currently the most studied agent among the five monoclonal antibodies for EoE or non-EoE EGIDs; hence, dupilumab was the drug of choice in this case.

Systemic Th2 disorders have been speculated to be responsible for the course of EoG. Moreover, IL-4, IL-5, and IL-13 pathway activation and increased expression of IL-4, IL-5, and IL-13 have been observed in the gastric tissues of patients with EoG [26]. Therefore, dupilumab may be effective owing to its pharmacological effect of suppressing the IL-4 and IL-13 pathways.

Aggravation of chronic eosinophilic pneumonia [20] or the development of eosinophilic granulomatosis with polyangiitis [22] have been observed as adverse effects of dupilumab administration. No evident adverse effects were observed in our patient’s case.

This report may have certain limitations. First, this study reports one case. Further research on dupilumab is required. Secondly, biopsies were not performed on the esophagus, small bowel, or colon, so the possibility of EoE, EoN, or EoC occurring together cannot be completely ruled out. However, the patient did not feel symptoms in the esophagus, and the endoscopic examination was normal, with no characteristic findings of EoE. In addition, there were no lower GI symptoms such as diarrhea that would suggest involvement of the small bowel or colon, and CT scans did not reveal thickening of the intestinal wall that would suggest enteritis. Thus, there were no symptoms or findings on examination that strongly suggested the presence of complications of esophageal, small bowel, or colon. Therefore, this case was diagnosed as EoG. Thirdly, the safety of dupilumab has not been fully evaluated in patients with EoG concomitant with severe asthma. In clinical trial NCT03678545, patients with poorly controlled asthma (ACT score < 20) were excluded. Therefore, studies that include patients with severe asthma are warranted.

## Conclusion

Dupilumab safely and effectively managed EoG concomitant with severe asthma. Therefore, dupilumab may be considered an effective therapeutic option for these patients.

## Data Availability

No datasets were generated or analysed during the current study.

## Abbreviations

EGIDs: Eosinophilic gastrointestinal diseases
EoG: Eosinophilic gastritis
IL: Interleukin
FeNO: Fractional exhaled nitric oxide
IgE: Immunoglobulin E
GI: Gastrointestinal
